# Deep learning based CETSA feature prediction cross multiple cell lines with latent space representation

**DOI:** 10.1038/s41598-024-51193-6

**Published:** 2024-01-22

**Authors:** Shenghao Zhao, Xulei Yang, Zeng Zeng, Peisheng Qian, Ziyuan Zhao, Lingyun Dai, Nayana Prabhu, Pär Nordlund, Wai Leong Tam

**Affiliations:** 1 Institute for Infocomm Research (I2R), A*STAR, Singapore, 138632 Singapore; 2 National University of Singapore (NUS), Singapore, 119077 Singapore; 3 Institute of Molecular and Cell Biology (IMCB), A*STAR, Singapore, 138632 Singapore; 4 The Second Clinical Medical College of Jinan University, The First Affiliated Hospital of Southern University of Science and Technology, Shenzhen People’s Hospital, Shenzhen, 518020 China; 5 Department of Oncology and Pathology, Karolinska Institutet, 171 77 Stockholm, Sweden; 6 Genome Institute of Singapore (GIS), A*STAR, Singapore, 138632 Singapore

**Keywords:** Computational biology and bioinformatics, Computational models

## Abstract

Mass spectrometry-coupled cellular thermal shift assay (MS-CETSA), a biophysical principle-based technique that measures the thermal stability of proteins at the proteome level inside the cell, has contributed significantly to the understanding of drug mechanisms of action and the dissection of protein interaction dynamics in different cellular states. One of the barriers to the wide applications of MS-CETSA is that MS-CETSA experiments must be performed on the specific cell lines of interest, which is typically time-consuming and costly in terms of labeling reagents and mass spectrometry time. In this study, we aim to predict CETSA features in various cell lines by introducing a computational framework called CycleDNN based on deep neural network technology. For a given set of *n* cell lines, CycleDNN comprises *n* auto-encoders. Each auto-encoder includes an encoder to convert CETSA features from one cell line into latent features in a latent space $\mathbb {Z}$. It also features a decoder that transforms the latent features back into CETSA features for another cell line. In such a way, the proposed CycleDNN creates a cyclic prediction of CETSA features across different cell lines. The prediction loss, cycle-consistency loss, and latent space regularization loss are used to guide the model training. Experimental results on a public CETSA dataset demonstrate the effectiveness of our proposed approach. Furthermore, we confirm the validity of the predicted MS-CETSA data from our proposed CycleDNN through validation in protein–protein interaction prediction.

## Introduction

In biology, cells are highly sophisticated and mutable intracellular spaces containing myriad interacting proteins that continuously transmit signals to actuate diverse cellular and biochemical processes. However, direct monitoring of the interaction status of native proteins with other biomolecules within intact cells has remained a challenging task until the introduction of the cellular thermal shift assay (CETSA)^1^.

CETSA utilizes the biophysical principles of ligand-induced thermal stabilization to directly monitor the interaction status of the target protein with ligand within intact cells^2^. In contrast to classical thermal shift assays (TSAs) used with purified proteins, CETSA can directly work with intact cells or lysates. In the classical CETSA assay, cell lysates or intact cells are heated to a range of temperatures, then cooled down and centrifuged to obtain the remaining soluble proteins in the supernatant for quantification. Protein quantification is originally carried out by Western blot in a targeted mode (commonly referred to as WB-CETSA), and later by using multiplexed mass spectrometry (MS), which is often referred to as MS-CETSA^3^. By determining the relative abundance of soluble proteins over a range of elevated temperatures, CETSA melting profiles provide insights into protein stability shifts induced by drug binding or other factors in the native cellular environment^4^.

MS-CETSA is widely used in the understanding of drug mechanisms of action (MoAs)^5–7^, the dissection of protein interaction dynamics in different cellular states^3,8,9^, the screening for potential ligands^10,11^, and so on. It should be noted that MS-CETSA can also be used to monitor protein–protein interactions (PPIs). PPIs, the highly specific physical contact between two or more protein molecules, not only have a physical and biochemical basis, but are also influenced by the cellular context^12^. We have reported the phenomenon of thermal proximity co-aggregation (TPCA), which is based on the observation that interacting proteins tend to co-aggregate upon thermal denaturation, as evidenced by similar melting curves over the temperature range^13^. However, the extent of relatively accurate correlations between CETSA features and PPIs has not been systematically investigated.

**Figure 1:**
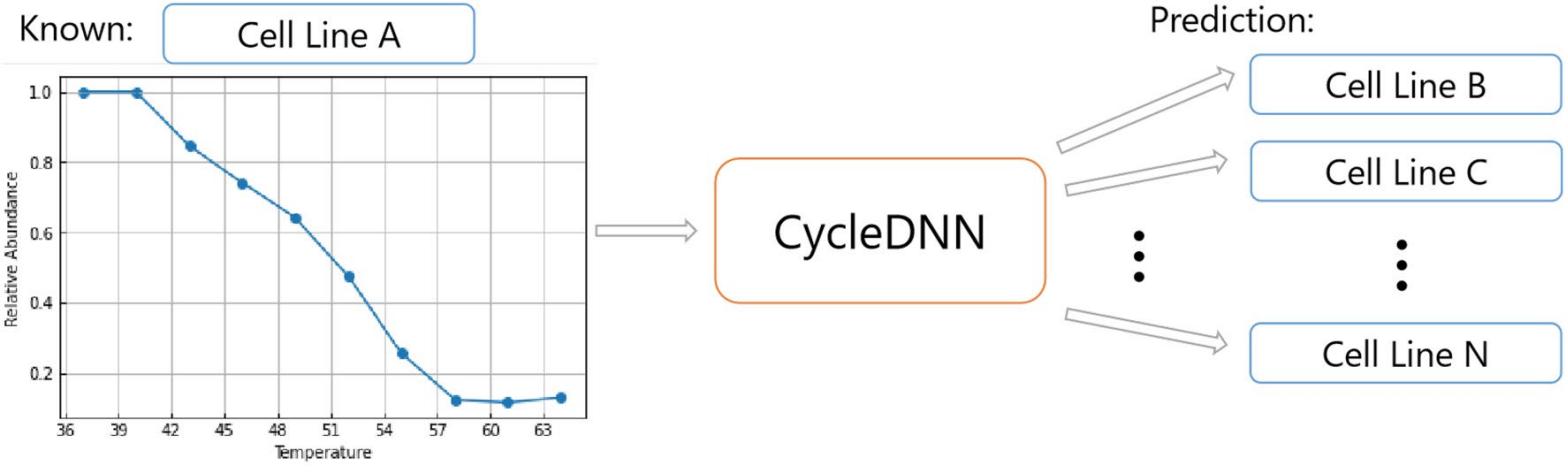
The diagram of the prediction of CETSA features across cell lines.

Despite its utility, there are still significant barriers to the large-scale application of MS-CETSA. A key challenge is the reliance on time-consuming and resource-intensive biological experiments to obtain protein melting curves of proteins for each cell line of interest. Generating complete MS-CETSA datasets across multiple cell lines is almost infeasible, given the current depth of mass spectrometry measurement. While some proteins are common across cell lines, others may only be present in specific contexts. To overcome this bottleneck, we develop a computational approach for predicting CETSA features across cell lines based on limited experimental data, as shown in Fig. 1. By extrapolating from one cell line to others, this predictive modeling aims to dramatically reduce the experimental burden and enable broader applications of MS-CETSA methodology.

In the field of machine learning, deep neural networks have driven revolutionary advances in various areas^14^, especially in computer vision. An important topic in computer vision is image-to-image translation, where the style of one image is transferred to another. Two influential models are pix2pix^15^ and CycleGAN^16^, which can perform robust image translation across different domains while preserving key texture and content attributes. These models inspire the developing of similar techniques for transferring features across different domains. Given that our proposed approach is primarily inspired by image-to-image translation techniques, other related technologies for cross-modality translation, e.g., non-image to image translation^17^, will not be discussed in this work.

Inspired by image translation techniques, we develop a novel deep learning framework called CycleDNN to predict CETSA features across cell lines. CycleDNN contains encoders $\{ E_1, E_2,\ldots E_n \}$ and decoders $\{ D_1, D_2\ldots D_n \}$ corresponding to cell lines $\{ C_1, C_2\ldots C_n \}$. Each encoder $E_i$ translates the CETSA features of cell line $C_i$ into a latent space $\mathbb {Z}$, and each decoder $D_i$ translates *Z* back into the CETSA feature in cell line $C_i$ $(i \in \{1, 2,\ldots n\})$. Any encoder and decoder can be paired to form an auto-encoder for predicting CETSA features from one cell line to another. Together they form the prediction from one cell line to another under the guidance of the prediction loss, the cycle-consistency loss, and the latent space regularization loss. Thus, our approach enables the reciprocal prediction of features across different cell lines. While our method is inspired by pix2pix and CycleGAN, it differs in that all our auto-encoders are constructed using deep neural networks (DNN) as opposed to generative adversarial networks (GAN)^18^. In addition, these auto-encoders have identical network architectures but operate with different parameters.

We have previously reported that MS-CETSA data could be used to predict PPI scores using the decision tree model, a classic machine learning model^19^. In this study, we further explore the PPI prediction from CETSA data and treat it as an evaluation metric to verify the efficiency of the proposed CycleDNN for CETSA feature prediction, i.e., whether the translated CETSA feature could also be adapted for PPI prediction. In our study, we use the predicted CETSA data by our proposed CycleDNN to predict the PPI scores and compare the performance with that of the experimental CETSA data taken from Tan et al.^13^. The PPI score prediction results further verify the effectiveness of our proposed method.

The preliminary results of this study have been reported in^20^. Significant changes have been made compared to our previous work. Firstly, we work out a novel training and testing framework that is computationally efficient and flexible for CETSA feature prediction across multiple cell lines. Secondly, we perform extensive experiments on multiple cell lines to verify the effectiveness of the proposed framework. Lastly, we included PPI prediction in an evaluation task and performed additional experiments to further verify the feasibility of the proposed framework. The main contributions of our research work are summarized as follows:

- We introduce a computational structure that utilizes a unique deep neural network model, CycleDNN, to convert CETSA characteristics among various cell lines. With only the CETSA features of a specific protein in a single cell line, our approach can accurately predict the CETSA features in other cell lines.
- By introducing the Z-hidden space, we adopt n encoders and n decoders corresponding to n cell lines to achieve the prediction of CETSA features of multiple cell lines. This reduces the complexity of the model from exponential to linear compared to the individual one-to-one prediction models.
- We perform extensive experiments on the public CETSA feature dataset and verify the effectiveness of our method. We further perform experiments using PPI predictions with predicted CETSA features from CycleDNN and achieve similar performance compared to experimental CETSA features.
- We publish the source codes^21^ of the implementation of the proposed method. Interested readers can use the source codes for their own biological feature predictions. We hope that this effort can motivate further exploration of deep neural networks for biological feature (e.g., CETSA) prediction.

## Background

To the best of our knowledge, we are the first ones to study and realize CETSA data prediction across cell lines. In this section, we will focus on related work in the field of computer vision and auto-encoders, both largely inspire our work in this study. Moreover, PPI is also closely related to our study.

### Image style transfer

Fueled by the progress in deep learning^22^ and generative adversarial networks^18^, significant strides have been made in the field of image style translation. A pioneering model in this domain is the neural algorithm introduced by Justin Johnson et al. in 2015^23^, which leverages VGG-19^24^ and posits that deep convolutions can distill content information, while shallow convolutions can extract style information.

Pix2pix^15^ and CycleGAN^16^ stand as prominent techniques for image-to-image translations, catering to paired and unpaired data respectively. Pix2pix refines the GAN architecture by integrating a conditional GAN model for a wide range of paired image translations. CycleGAN, conversely, is an advancement of the GAN architecture that caters to unpaired data, involving the parallel training of dual generator and discriminator models to create a cyclic route. Within this context of mutual translation of paired attributes, we borrow the concept of “Consistency” from CycleGAN. This concept suggests that the output from the second generator can serve as input to the first generator, and the outcome should correspond to the input to the second generator, and vice versa. Similarly, our CycleDNN method constructs a “cycle” to ensure that when a protein in cell line *A* with all its features is processed through Encoder $E_A$, Decoder $D_B$, Encoder $E_B$, and Decoder $D_A$, the output corresponds to the input protein features in cell line *A*.

### Auto-encoders

The idea of the latent space $\mathbb {Z}$ is inspired by auto-encoders. Auto-encoders are a type of algorithm to learn a hidden “informative” representation of the data, which was first proposed by Rumelhart et al.^25^. With the help of the nonlinear feature extraction ability of the deep neural network, auto-encoders can obtain a good data representation, and the performance of the autoencoder is better than linear methods such as principal component analysis (PCA)^26^. In this study, the hidden “informative” representation can be considered as the common latent space $\mathbb {Z}$, i.e., latent representations of the same protein that does not change when the cell line changes. However, the difference between auto-encoders and our method is that our goal is mutual predictions rather than reconstruction.

In contrast to CNNs, GANs, and other models commonly used in image transfer models, the main body of our model adopts the structure of the deep neural network (DNN), also known as Multilayer Perceptron (MLP) and artificial neural network (ANN). It is the most classical deep learning model, developed on the basis of the single-layer perceptron. It is also the most common model for auto-encoders and the most suitable model for the CETSA data.

### PPI prediction

The research on PPI is mainly divided into two categories. The first one is using experimental methods, such as yeast two-hybrid screening^27^, nucleic acid programmable protein array (NAPPA)^28^, affinity purification–mass spectrometry (AP–MS)^29^, correlated mRNA expression profile^30^, synthetic lethal analysis^31^and so on. However, this kind of method is normally time-consuming and expensive. Moreover, experimental results often show notable inter- or intra-variance. This leads to the second type of method that uses computational models and other properties of proteins to predict PPIs.

The research of PPI prediction through computational models has developed particularly rapidly in recent years, mainly due to advances in machine learning and deep learning. Various new methods of deep learning, machine learning and other statistical methods are combined with various protein data to produce various new prediction methods for PPI, such as network-based models^32^, sequence-based models^33^, structure-based models^34^, genomic-based models^35^and so on. But so far, no one except our group has tried to use CETSA data to predict PPI^19^.

## Methodology

### CETSA data

The CETSA data used in this study is from Tan et al. in 2018^13^, which consists of multiple cell lines. Each cell line consists of more than seven thousand proteins, and each protein contains 10 features from 10 temperatures. For a pair of cell lines, there are certain common proteins with CETSA features in both cell lines, while for other proteins, their CETSA features exist only in one cell line. We train the cycleDNN model based on the common proteins, the trained model can be used to predict the CETSA features from one cell line to another for those proteins that have CETSA features in only one cell line.

### CycleDNN for two cell lines

Deep neural networks^22^ are extensively employed in both classification and generative models across various fields such as computer vision and natural language processing. These networks exhibit greater expressiveness and feature extraction capabilities compared to perceptrons. The fundamental architecture of these networks typically encompasses an input layer, multiple hidden layers, and an output layer. Each node within the network primarily executes a blend of a linear operation and a nonlinear activation function.

**Figure 2:**
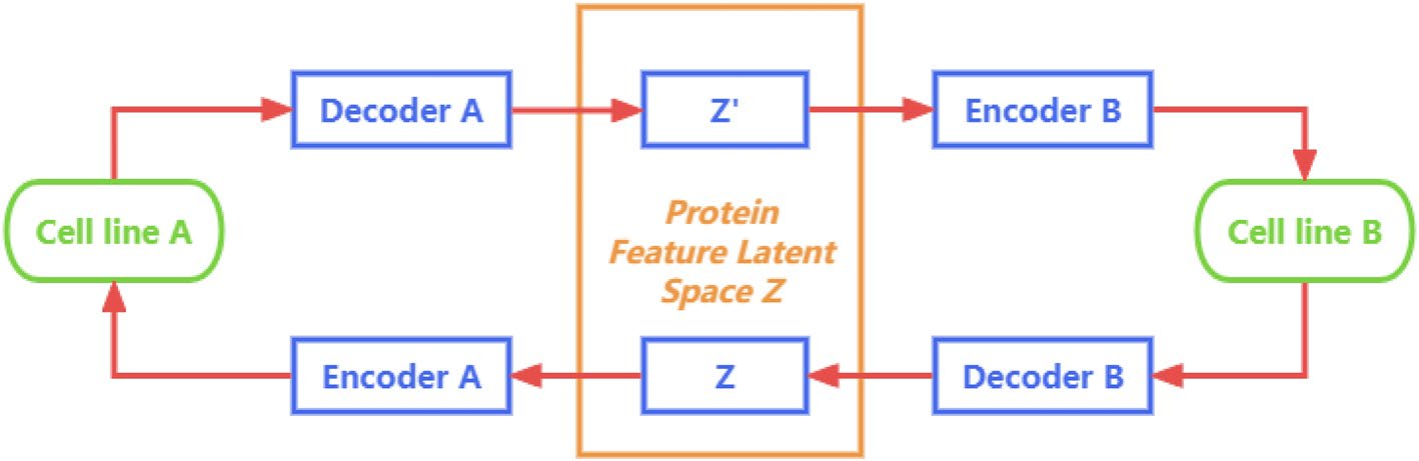
An illustration of utilizing CycleDNN for the transference of CETSA features between cell line *A* and cell line *B*. *Z* and $Z'$ are kept nearly identical to establish a shared protein latent space.

Our initial step involves the construction of a model that enables pairwise data prediction across two distinct cell lines. In this scenario, CycleDNN is primarily composed of two encoders and two decoders. For a protein that concurrently exists in two cell lines (for instance, HCT116 and HEK293T, designated as cell lines A and B), we utilize two encoders, $E_A$ and $E_B$, to transform the 10-dimensional CETSA features into a shared latent space $\mathbb {Z}$, which consists of 5000-dimensional latent features. Decoders $D_A$ and $D_B$ are then employed to revert $\mathbb {Z}$ back to the CETSA data of cell lines A and B, respectively. During the training phase, we simultaneously train both sets of encoders and decoders. Figure 2 provides a comprehensive diagram of CycleDNN for the prediction of CETSA features between the two cell lines.

**Figure 3:**
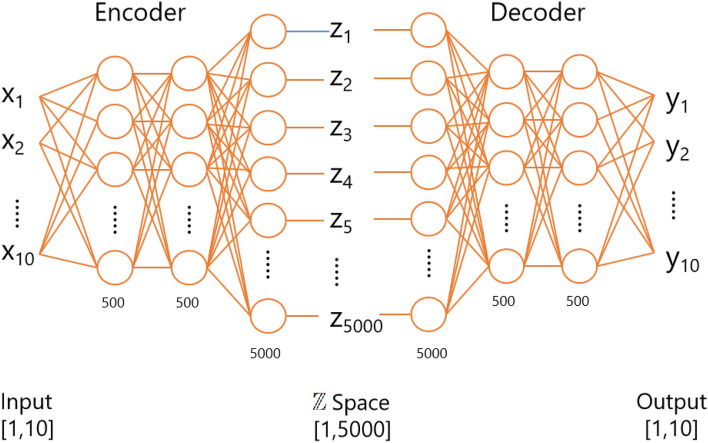
Architecture of the encoder and decoder within CycleDNN.

**Table 1:** CycleDNN architecture: detailed parameters and characteristics of encoder and decoder layers.

Model	Layer	Type	Input size	Output size	Activation function
Encoder	Layer1	Fully connected	10	500	ReLU
	Layer2	Fully connected	500	500	ReLU
	Layer3	Fully connected	500	5000	ReLU
Decoder	Layer1	Fully connected	5000	500	ReLU
	Layer2	Fully connected	500	500	ReLU
	Layer3	Fully connected	500	10	

Both the encoder and the decoder are constructed using DNNs. The two encoders, $E_A$ and $E_B$, as depicted in Fig. 2, possess identical network structures. However, it’s crucial to note that they do not share parameters. Similarly, the two decoders, denoted as $D_A$ and $D_B$, also maintain the same network structure but do not share parameters. The encoder’s network structure primarily consists of three fully connected layers, each incorporating a linear operation and a linear rectification activation function (ReLU). Additionally, a dropout layer is included to mitigate overfitting. The decoder’s structure mirrors that of the encoder but is assembled in the reverse order. Figure 3 illustrates the integrated encoder and decoder architecture in CycleDNN transitioning from cell line A to cell line B. Detailed networks’ parameters are shown in Table 1.

### CycleDNN for multiple cell lines

**Figure 4:**
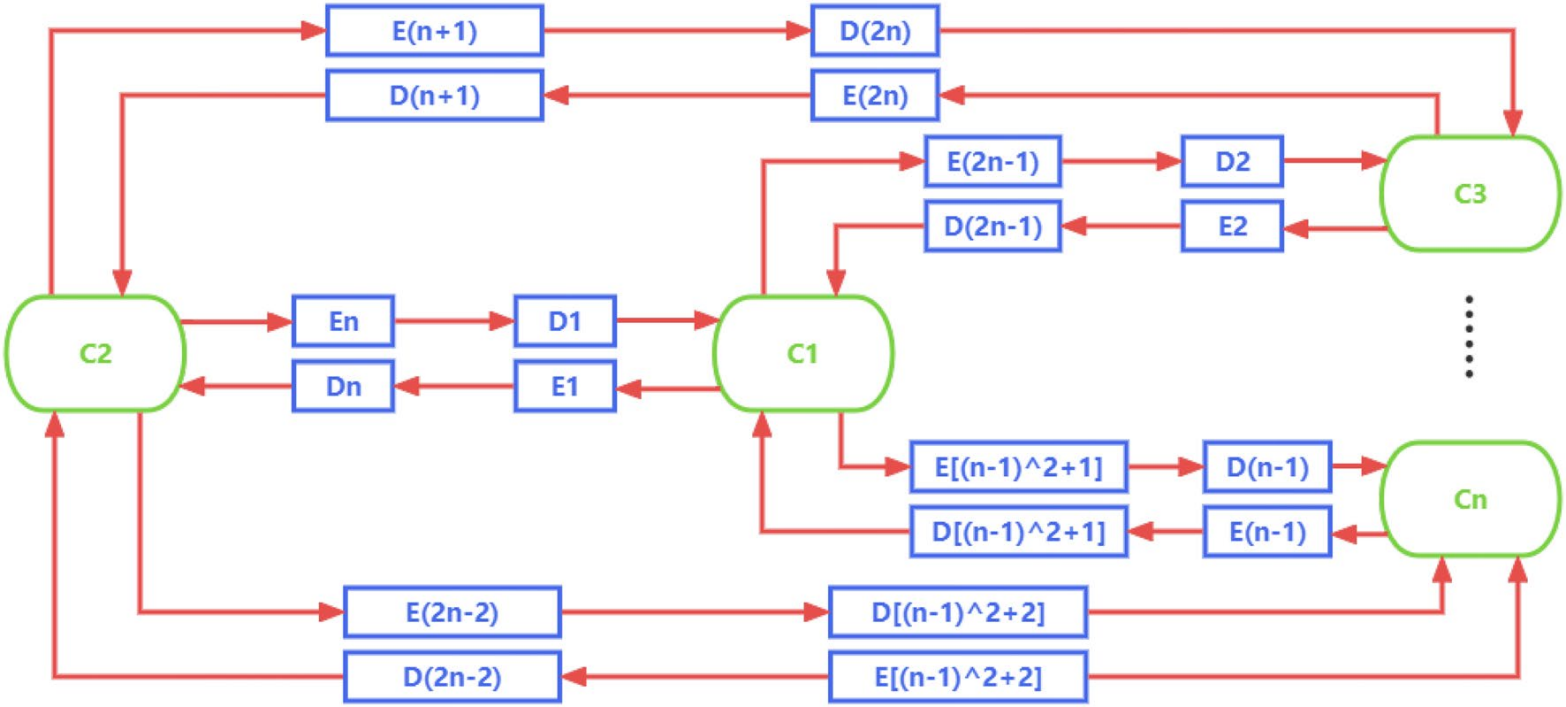
The diagram of standard model with $n(n-1)$ encoders and $n(n-1)$ decoders for cell lines $\{ C_1, C_2, \dots , C_n \}$.

In this subsection, we make an effort to generalize the feature prediction by CycleDNN from two cell lines to multiple cell lines. The key to the generalization is the common latent space $\mathbb {Z}$. If we directly use the deep neural network to achieve prediction in any two cell lines, we need to train $n(n-1)$ neural networks for cell lines $\{ C_1, C_2\ldots C_n \}$. Assuming that each deep neural network can be decomposed into an encoder and a decoder, and a total of $n(n-1)$ encoders and $n(n-1)$ decoders are required, as shown in Fig. 4.

Such a model has a significant disadvantage: as the number of cell lines grows, the number of networks that need to be trained will grow exponentially, which is highly complex and expensive. To overcome this disadvantage, we introduce the common latent space $\mathbb {Z}$ and redesign the structure of CycleDNN for the CETSA feature prediction of various cell lines.

**Figure 5:**
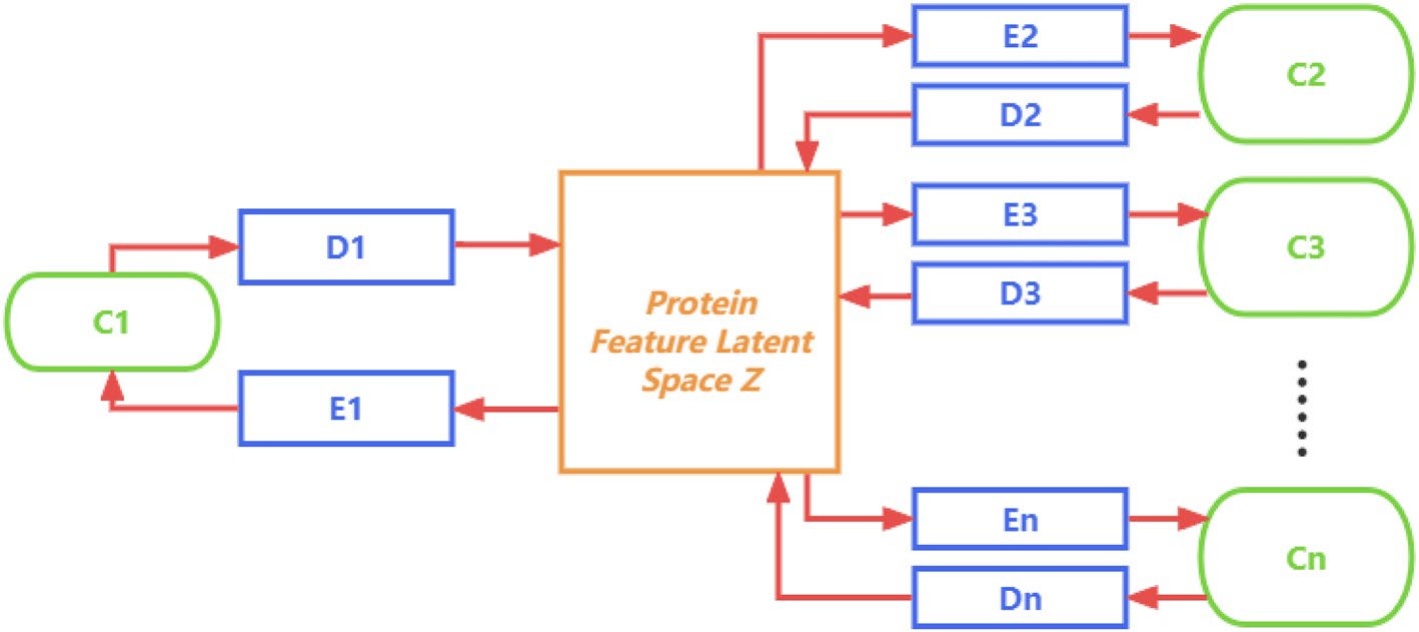
The diagram of CycleDNN with *n* encoders, *n* decoders and latent space $\mathbb {Z}$ for cell lines $\{ C_1, C_2, \ldots , C_n \}$.

The common latent space $\mathbb {Z}$ represents latent representations of the same protein that does not change when the cell line changes. So the CETSA data of any cell line can be mapped to the common latent space $\mathbb {Z}$ after being encoded by the encoder of the corresponding cell line. Moreover, any decoder can decode the $\mathbb {Z}$ to the CETSA features in the corresponding cell line. Based on the common latent space $\mathbb {Z}$, we design a new CycleDNN structure, as shown in Fig. 5.

In the new CycleDNN structure, we only need *n* encoders and *n* decoders for cell lines $\{ C_1, C_2, \ldots , C_n \}$. This new structure reduces the complexity of the model requirement from exponential to linear, which greatly reduces the parameters and training cost of the model. Our method using this structure can be generalized to any number of cell lines efficiently and flexibly. We constrain the common latent space $\mathbb {Z}$ by designing loss functions. In the experiment, we perform the prediction of CETSA features across five human cell lines A375, HCT116, HEK293T, HL60, and MCF7.

### Loss functions

As depicted in Fig. 5, we can discern the following mapping relationships between different cell lines: (1) Encoder $E_i$: $C_i$ $\rightarrow$ *Z*; (2) Decoder $D_i$: *Z* $\rightarrow$ $C_i$. Through *Z*, we can derive the mapping function from Encoder $E_i$ to Decoder $D_j$ as $F_{i,j}: C_i\rightarrow C_j$.

In terms of loss function design, we consider three varieties. The first kind is the mean square error (MSE) loss, which measures the discrepancy between the predicted data and the ground truth, referred to as prediction loss. This category of loss function encompasses the loss incurred when predicting cell line *B* from cell line *A* and vice versa.

1 $$\begin{aligned} L_1 =\frac{\sum _{i, j=1}^n MSE(F_{i,j} (C_i), C_j)}{n}. \end{aligned}$$

The second category of the loss function is the Cycle-Consistency Loss, a concept inspired by CycleGAN. Given the mapping functions $F_{i,j}: C_i\rightarrow C_j$, it’s logical to assume that if we generate cell line $C_j$ from cell line $C_i$ using the mapping function $F_{i,j}$, we should be able to reconstruct cell line $C_i$ from the generated cell line $C_j$ using the mapping function $F_{j,i}$. In other words, $F_{j,i} (F_{i,j} (C_i)) \approx C_i$. Hence, the second component of the loss function can be articulated as:

2 $$\begin{aligned} L_2 = \frac{\sum _{i, j=1}^n MSE(F_{j,i} (F_{i,j} (C_i)), C_i)}{n}. \end{aligned}$$

The third category of loss function ensures consistency within the latent space Z, referred to as latent space regularization loss. This loss function guarantees that the underlying representations in the latent space Z of different cell lines of homogeneous proteins are approximately similar, which is critical for the successful prediction of CETSA signatures across cell lines. These potential representations in high-dimensional space should capture essential features of the protein that do not change because the protein is present in different cell lines. $Z_i$ is the potential representation of the protein obtained by the encoder $E_i$ of cell line $C_i$, and $Z_k$ is a potential representation of the same protein but from a different cell line $C_k$. n is the number of cell lines we adopted. Therefore, to maintain consistency in the output of encoders $E_{i}$ and $E_k$, a mean-squared loss among $Z_i$ and $Z_k$ is implemented as follows:

3 $$\begin{aligned} L_3 = \frac{\sum _{k=1}^n \sum _{i=1}^n MSE(Z_{i}, Z_{k})}{n^2}. \end{aligned}$$

Ultimately, the aforementioned three types of losses are amalgamated with distinct coefficients $\alpha _1$, $\alpha _2$, and $\alpha _3$, which are optimized empirically. The cumulative loss function of the proposed method is expressed as follows:

4 $$\begin{aligned} L = \alpha _1 L_1+\alpha _2 L_2 +\alpha _3 L_3. \end{aligned}$$

## Performance evaluation and discussion

### Datasets description

The dataset we utilized originates from the experimental data of Tan et al. in 2018^13^. We adopted the protein melting data from A375, HCT116, HEK293T, HL60, and MCF7 intact cell CETSA experiments as the CETSA features of different cell lines for mutual prediction. These proteins’ CETSA melting curves were downloaded from Tan et al. in 2018. The data file “tabless1_to_s27.zip” includes 27 CETSA data tables. Among these 27 tables, we selected five tables: S19, S20, S21, S22, and S23, which correspond to the five intensive cell CETSA experiments of A375, HCT116, HEK293T, HL60, and MCF7. We select the column attributes in these tables as T37, T40, T43, T46, T49, T52, T55, T58, T61 and T64. The ten columns serve as input to our model, which are the specific values of the protein melting curve.

The CETSA features for these five cell lines encompass relative abundance data for 8101, 7599, 7945, 7448, and 7790 proteins at ten distinct temperatures (37 $^{\circ }$C, 40 $^{\circ }$C, 43 $^{\circ }$C, 46 $^{\circ }$C, 49 $^{\circ }$C, 52 $^{\circ }$C, 55 $^{\circ }$C, 58 $^{\circ }$C, 61 $^{\circ }$C, and 64 $^{\circ }$C), which have been standardized. As common protein data are necessary for z-space prediction, we use the intersection of A375, HCT116, HEK293T, HL60, and MCF7, which includes common data for a total of 4860 proteins, as the benchmark to evaluate the proposed method across multiple cell lines. These CETSA data are fed into the neural network. Finally, the datasets are randomly split into training and test sets at a ratio of 70–$30\%$.

The PPI dataset Bioplex 3.0^36^ is adopted as ground truth in evaluation. PPI scores between two proteins of Bioplex 3.0 are a normalized value between [0, 1]. A bigger score indicates a higher probability of interaction between the two proteins. There are 25,485 protein pairs for cell line HCT116 and 41,490 protein pairs for cell line HEK293T in the CETSA dataset. Since the PPI data of HEK293T in Bioplex is relatively more comprehensive, we only use the PPI data of HEK293T for training and testing.

### Experimental setup

#### Metrics

To validate the efficacy of our model, we employ the following evaluation metrics. Mean square error (MSE), mean absolute percentage error (MAPE), mean absolute error (MAE), R-squared (${\hbox {R}}^{2}$), and Pearson correlation coefficient (PCC) are frequently used as evaluation metrics in regression models. Assuming the model’s input is *X*, the predicted value is $y'$, the actual value is *y*, and the mean value of *y* and $y'$ are $\bar{y}$ and $\bar{y'}$, the expressions for these evaluation parameters are as follows:

5 $$\begin{aligned} MSE(y,y')= & {} \frac{1}{n} \sum _{i=1}^n (y_i-y'_i)^2, \end{aligned}$$

6 $$\begin{aligned} MAPE(y,y')= & {} \frac{100\%}{n} \sum _{i=1}^n \left| \frac{(y_i-y'_i)^2}{y_i} \right| , \end{aligned}$$

7 $$\begin{aligned} MAE(y,y')= & {} \frac{1}{n} \sum _{i=1}^n\left| y_i-y'_i\right| . \end{aligned}$$

8 $$\begin{aligned} R^2(y, y')= & {} 1 - \frac{\sum _{i=1}^n (y_i - y'_i)^2}{\sum _{i=1}^n (y_i - \bar{y})^2}, \end{aligned}$$

9 $$\begin{aligned} PCC(y, y')= & {} \frac{\sum _{i=1}^n (y_i - \bar{y})(y'_i - \bar{y'})}{\sqrt{\sum _{i=1}^n (y_i - \bar{y})^2 \sum _{i=1}^n (y'_i - \bar{y'})^2}}, \end{aligned}$$

#### Implementation details

In this study, we adopt the PyTorch library to implement our model and conduct CETSA feature prediction experiments on the NVIDIA platform with GeForce GTX TITAN X GPUs. We train the model on the training set and evaluate the performance on the test set. During training, CETSA data is randomly shuffled. Each model is trained for a total of 4000 epochs on 1 GPU with a total batch size of 128. All models are trained from scratch and are optimized using stochastic gradient descent with momentum at 0.95 and weight decay of $1e-5$. The base learning rate is 0.01 and declined by $5\%$ every 500 iterations. The dropout rate is set to 0.3 to improve the robustness. The hyper-parameters $\alpha _{1\sim 3}$ are 1, 0.01, and 1, respectively.

#### Training details

At each epoch, CETSA features in each cell line are translated to all cell lines. In addition, CETSA features in each cell line are reconstructed after prediction to keep cycle-consistency. For example, when we only consider the case of three cell lines, at each epoch, the features in cell line A are translated to cell lines B and C through the corresponding encoder and decoders. After prediction, the predicted CETSA features are reconstructed from cell lines B and C back to the CETSA features of cell line A. The same training procedure is repeated for cell lines B and C.

In the training process across five cell lines, our strategy involved sequentially training the model to predict CETSA features from cell line A to cell line A...E, followed by cell line B to cell line A...E, and so forth, until cell line E. The training was set to stop if there were no observed improvements in performance for 300 consecutive epochs. Additionally, the maximum number of training epochs was capped at 5000.

In line with the implementation details, our model obtained convergence after 4000 epochs in training, culminating in optimal performance. This process was completed in a span of 4.5 h within our test environment. Additionally, during the training of two distinct cell lines, optimal results were achieved with just 1.6 h of training. This duration of time consumption aligns closely with the scale of our model.

#### PPI prediction evaluation

Machine learning methods are widely used in various fields of bioinformatics. We adopt a decision tree as our model to predict PPI scores between protein pairs based on the protein’s CETSA features. The 5-fold cross-validation approach is applied to the decision tree model, where the ratio of the training set and test set is 4:1. In comparison, we use the CETSA experimental data and the CETSA data predicted by CycleDNN to train the PPI prediction models, respectively. If the two prediction models achieve similar performance, it indicates the validity of the predicted CETSA features from our proposed CycleDNN.

### Numerical results

#### Two cell lines (A375 and HCT116)

**Table 2:** Performance of CycleDNN between cell line *A* (A375) and cell line *B* (HCT116).

Transfer	MSE	MAPE	MAE	$R^{2}$	*PCC*
$A\rightarrow A$	0.00058	$2.225\%$	$6.64\times 10^{-5}$	0.98765	0.99381
$A\rightarrow B$	0.01232	$13.971\%$	$4.61\times 10^{-4}$	0.89773	0.94750
$B\rightarrow A$	0.01805	$15.950\%$	$5.68\times 10^{-4}$	0.88773	0.94221
$B\rightarrow B$	0.00048	$2.026\%$	$5.89\times 10^{-5}$	0.98606	0.99301

The main results of this study with two cell lines are listed in Table 2. According to the results, the MSE, MAPE, MAE, $R^{2}$ and PCC of our models reach 0.01232, $13.971\%$, $4.61\times 10^{-4}$, 0.89773 and 0.94750 in the prediction from the A375 cell line to the HCT116 cell line. It also works in the prediction from the HCT116 cell line to the A375 cell line, which reaches 0.01805, $15.950\%$, $5.68\times 10^{-4}$, 0.88773 and 0.94221 in MSE, MAPE, MAE, $R^{2}$ and PCC. Moreover, CETSA feature predictions of the original cell lines, as $A\rightarrow A$ and $B\rightarrow B$ shown in Table 2, are much more precise than those of other cell lines. This phenomenon also exists in subsequent experiments. Since we are the first to realize automatic CETSA feature prediction across cell lines, there are no existing research methods to compare.

#### Multiple cell lines

**Table 3:** Performance of CycleDNN between cell line *A* (A375), cell line *B* (HCT116) and cell line *C* (HEK293T).

Transfer	MSE	MAPE	MAE	$R^{2}$	*PCC*
$A\rightarrow A$	0.00083	$2.699\%$	$8.77\times 10^{-5}$	0.98716	0.99356
$A\rightarrow B$	0.01163	$14.039\%$	$4.63\times 10^{-4}$	0.89963	0.94851
$A\rightarrow C$	0.00635	$12.197\%$	$3.22\times 10^{-4}$	0.95264	0.97605
$B\rightarrow A$	0.01798	$15.798\%$	$5.64\times 10^{-4}$	0.88885	0.94280
$B\rightarrow B$	0.00058	$2.710\%$	$8.16\times 10^{-5}$	0.98502	0.99248
$B\rightarrow C$	0.00805	$14.227\%$	$3.85\times 10^{-4}$	0.94284	0.97087
$C\rightarrow A$	0.01464	$12.832\%$	$4.94\times 10^{-4}$	0.90679	0.95234
$C\rightarrow B$	0.01342	$14.808\%$	$5.02\times 10^{-4}$	0.89142	0.94416
$C\rightarrow C$	0.00039	$2.749\%$	$6.65\times 10^{-5}$	0.99142	0.99570

**Table 4:** Performance of CycleDNN between cell line *A* (A375), cell line *B* (HCT116), cell line *C* (HEK293T), cell line *D* (HL60) and cell line *E* (MCF7).

*Transfer*	*MSE*	*MAPE*	*MAE*	$R^{2}$	*PCC*
$A\rightarrow A$	0.00138	$3.953\%$	$1.33\times 10^{-4}$	0.98614	0.99304
$A\rightarrow B$	0.01155	$14.253\%$	$4.68\times 10^{-4}$	0.90006	0.94874
$A\rightarrow C$	0.00632	$11.589\%$	$3.27\times 10^{-4}$	0.95017	0.97482
$A\rightarrow D$	0.02186	$20.461\%$	$7.39\times 10^{-4}$	0.78524	0.88650
$A\rightarrow E$	0.01595	$13.465\%$	$4.00\times 10^{-4}$	0.87805	0.93713
$B\rightarrow A$	0.01811	$16.480\%$	$5.78\times 10^{-4}$	0.88930	0.94305
$B\rightarrow B$	0.00099	$3.852\%$	$1.31\times 10^{-4}$	0.98312	0.99153
$B\rightarrow C$	0.00832	$14.506\%$	$3.91\times 10^{-4}$	0.94156	0.97039
$B\rightarrow D$	0.02164	$20.260\%$	$7.30\times 10^{-4}$	0.79079	0.88944
$B\rightarrow E$	0.01491	$15.620\%$	$4.42\times 10^{-4}$	0.89518	0.94620
$C\rightarrow A$	0.01476	$12.793\%$	$4.95\times 10^{-4}$	0.90698	0.95243
$C\rightarrow B$	0.01290	$13.939\%$	$4.90\times 10^{-4}$	0.89288	0.94493
$C\rightarrow C$	0.00066	$3.886\%$	$1.12\times 10^{-4}$	0.99138	0.99568
$C\rightarrow D$	0.02285	$21.408\%$	$7.46\times 10^{-4}$	0.77768	0.88201
$C\rightarrow E$	0.01510	$13.911\%$	$3.90\times 10^{-4}$	0.89264	0.94481
$D\rightarrow A$	0.02969	$28.057\%$	$8.18\times 10^{-4}$	0.81966	0.90541
$D\rightarrow B$	0.02294	$21.548\%$	$6.82\times 10^{-4}$	0.83296	0.91271
$D\rightarrow C$	0.01501	$24.768\%$	$5.84\times 10^{-4}$	0.89499	0.94605
$D\rightarrow D$	0.00092	$3.205\%$	$1.48\times 10^{-4}$	0.98172	0.99082
$D\rightarrow E$	0.02507	$25.580\%$	$6.13\times 10^{-4}$	0.82541	0.90855
$E\rightarrow A$	0.02183	$18.032\%$	$6.10\times 10^{-4}$	0.86737	0.93162
$E\rightarrow B$	0.01756	$16.577\%$	$5.64\times 10^{-4}$	0.86919	0.93247
$E\rightarrow C$	0.00932	$13.892\%$	$3.73\times 10^{-4}$	0.93168	0.96542
$E\rightarrow D$	0.02349	$21.086\%$	$7.51\times 10^{-4}$	0.77419	0.88009
$E\rightarrow E$	0.00092	$3.710\%$	$1.05\times 10^{-4}$	0.98335	0.99164

The main results of this study with three cell lines and five cell lines are listed in Tables 3 and 4, respectively. It can be seen from the experimental data that our model can be effectively applied no matter which two cell lines are used for CETSA feature prediction. The experimental results in Tables 3 and 4 with three and five cell lines are also similar to those with two cell lines. It further verifies the validity of our method’s design for multiple cell lines.

As can be seen from Tables 3 and  4, in the prediction across different cell lines, most results of CETSA feature prediction achieved the MSE below 0.002, the MAPE below 20%, the MAE below 0.001, the $R^{2}$ above 0.75 and the PCC above 0.88. This illustrates the overall effectiveness of our method. Meanwhile, the quality of the prediction results differs among cell lines. The best performance of the CETSA feature prediction with three and five cell lines is from cell line A (A375) to cell line C (HEK293T). In Table 4, it reaches 0.00632, $11.589\%$, $3.27\times 10^{-4}$, 0.95017 and 0.97482 in MSE, MAPE, MAE, $R^{2}$ and PCC. Moreover, its MSE, MAPE, MAE, $R^{2}$ and PCC also reach 0.00635, $12.197\%$, $3.22\times 10^{-4}$, 0.95015 and 0.97281 in Table 3.

In addition, adding more cell lines to our method can partially improve the performance of our model. From the comparison of Tables 3 and 4, we can see that the accuracy of some predictions is improved. For example, the prediction precision from cell line A (A375) to cell line C (HEK293T) in the model of five cell lines is better than that in the model of three cell lines. Its MSE, MAPE, and PCC are improved from 0.00635, $12.197\%$, and 0.97281 to 0.00632, $11.589\%$ and 0.97482. This indicates that adding more cell lines may further improve the accuracy of extracting information from the latent *Z* space.

### Ablation study

In this section, we explore the performance of different loss functions in the proposed method by conducting ablation experiments, including cell lines A375, HCT116, and HEK293T. As mentioned in the methodology section, we propose CycleDNN with prediction loss $L_1$, cycle-consistency loss $L_2$, and latent space regularization loss $L_3$. We explore all these variants quantitatively.

Table 5 shows the MSE, MAPE, and MAE results of different variants of the proposed network. Comparing all variants with our complete proposed model, it can be seen that all of the loss functions contribute to the performance, while $L_1$ plays the most important role. CycleDNN, by employing all three loss functions, amalgamates the benefits of each loss function, and the optimal performance is achieved through coefficient optimization. These experimental comparisons underscore the effectiveness of each of the three loss functions in our proposed method, thereby validating the design of our method. Notably, CycleDNN with $\mathbb {Z}$ space proves to be valuable. From a biological standpoint, the same protein, though encoded from different cell lines via the corresponding encoder, should possess common features in the latent space $\mathbb {Z}$.

**Table 5:** Performance of CycleDNN with different loss between cell line *A* (A375), cell line *B* (HCT116) and cell line *C* (HEK293T).

Loss	MSE ($A\rightarrow B$)	MAPE ($A\rightarrow B$)	MAE ($A\rightarrow B$)	$R^{2}$ ($A\rightarrow B$)	*PCC* ($A\rightarrow B$)
w/o L3	0.01217	$14.292\%$	$4.82 \times 10^{-4}$	0.89922	0.94829
w/o L2	0.01202	$14.184\%$	$4.65 \times 10^{-4}$	0.89950	0.94840
w/o L1	0.04295	$41.50\%$	$1.09 \times 10^{-3}$	0.68385	0.82718
L1+L2+L3	$\varvec{0.01163}$	$\varvec{14.039\%}$	$\mathbf{4.63} \times {\textbf{10}}^{\mathbf{-4}}$	$\varvec{0.89963}$	$\varvec{0.94851}$

Loss	MSE ($A\rightarrow C$)	MAPE ($A\rightarrow C$)	MAE ($A\rightarrow C$)	$R^{2}$ ($A\rightarrow C$)	*PCC* ($A\rightarrow C$)
w/o L3	0.00647	$11.960\%$	$3.27\times 10^{-4}$	0.95215	0.97581
w/o L2	0.00653	$\varvec{11.898\%}$	$3.31\times 10^{-4}$	0.95231	0.97588
w/o L1	0.02895	$46.296\%$	$9.21\times 10^{-4}$	0.79577	0.89225
L1+L2+L3	$\varvec{0.00635}$	$12.197\%$	$\mathbf{3.22}\times {\textbf{10}}^{\mathbf{-4}}$	$\varvec{0.95264}$	$\varvec{0.97605}$

Loss	MSE ($B\rightarrow A$)	MAPE ($B\rightarrow A$)	MAE ($B\rightarrow A$)	$R^{2}$ ($B\rightarrow A$)	*PCC* ($B\rightarrow A$)
w/o L3	0.01876	$15.822\%$	$5.68\times 10^{-4}$	0.88844	0.94258
w/o L2	$\varvec{0.01792}$	$15.814\%$	$5.65\times 10^{-4}$	0.88877	0.94275
w/o L1	0.05142	$47.022\%$	$1.20\times 10^{-3}$	0.68391	0.82715
L1+L2+L3	0.01798	$\varvec{15.798\%}$	$\mathbf{5.64}\times {\textbf{10}}^{\mathbf{-4}}$	$\varvec{0.88885}$	$\varvec{0.94280}$

Loss	MSE ($B\rightarrow C$)	MAPE ($B\rightarrow C$)	MAE ($B\rightarrow C$)	$R^{2}$ ($B\rightarrow C$)	*PCC* ($B\rightarrow C$)
w/o L3	0.00812	$14.114\%$	$3.80\times 10^{-4}$	0.94261	0.97091
w/o L2	$\varvec{0.00800}$	$\varvec{13.971\%}$	$3.84\times 10^{-4}$	0.94253	0.97102
w/o L1	0.02890	$46.028\%$	$9.25\times 10^{-4}$	0.79475	0.89158
L1+L2+L3	0.00805	$14.227\%$	$\mathbf{3.85}\times {\textbf{10}}^{\mathbf{-4}}$	$\varvec{0.94284}$	$\varvec{0.97087}$

Loss	MSE ($C\rightarrow A$)	MAPE ($C\rightarrow A$)	MAE ($C\rightarrow A$)	$R^{2}$ ($C\rightarrow A$)	*PCC* ($C\rightarrow A$)
w/o L3	0.01486	$13.181\%$	$\mathbf{4.90}\times {\textbf{10}}^{\mathbf{-4}}$	0.90602	0.95193
w/o L2	0.01489	$13.267\%$	$5.00\times 10^{-4}$	0.90654	0.95221
w/o L1	0.05143	$47.008\%$	$1.20\times 10^{-3}$	0.68321	0.82664
L1+L2+L3	$\varvec{0.01464}$	$\varvec{12.832\%}$	$4.94\times 10^{-4}$	$\varvec{0.90679}$	$\varvec{0.95234}$

Loss	MSE ($C\rightarrow B$)	MAPE ($C\rightarrow B$)	MAE ($C\rightarrow B$)	$R^{2}$ ($C\rightarrow B$)	*PCC* ($C\rightarrow B$)
w/o L3	0.01359	$\varvec{14.329\%}$	$4.90\times 10^{-4}$	0.89027	0.94355
w/o L2	0.01345	$14.711\%$	$\mathbf{4.75}\times {\textbf{10}}^{\mathbf{-4}}$	0.89069	0.94377
w/o L1	0.04292	$47.008\%$	$1.08\times 10^{-3}$	0.68156	0.82565
L1+L2+L3	$\varvec{0.01342}$	$14.808\%$	$5.02\times 10^{-4}$	$\varvec{0.89142}$	$\varvec{0.94416}$

L1 = prediction loss, L2 = cycle consistency loss, L3 = latent space regularization loss.

### Protein–protein prediction

**Figure 6:**
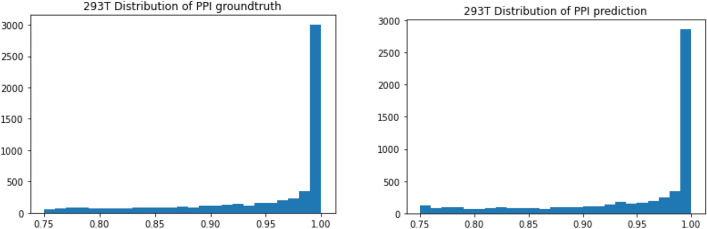
The distributions of the ground-truth (left) and predictions (right) of PPI scores in cell line HEK293T.

In this part, we use the CETSA features of 4860 proteins of HEK293T predicted from cell line A375 through trained CycleDNN as the input to the decision tree model. Our predicted input corresponds to 21,536 protein interaction pairs in BioPlex 3.0^37^. In the results of PPI prediction using a decision tree, our predicted CETSA data of cell line HEK293T obtained an MAE evaluation of 0.072198, which is very close to the MAE of 0.070726 obtained from the experimental CETSA data. Moreover, as shown in Fig. 6, the shape of histograms for prediction and ground truth are quite similar, which indicates that the predicted PPI scores match the ground truth PPI scores very well. This further verifies the effectiveness of our prediction model CycleDNN in the applications of CETSA data.

### Advantages and limitations

In our method, an encoder contains 2.761*M* parameters, and the computational cost of that is 5.510*M* FLOPs. Moreover, a decoder in our method contains 2.755*M* parameters, and the computational cost of that is 2.756*M* FLOPs.

The emergence of CycleDNN greatly reduces the workload of CETSA biochemical experiments. For a typical task, we need to know the CETSA value of a certain protein in *n* cell lines. If we rely solely on experiments, we will have to repeat *n* times of CETSA biochemical experiments in *n* cell lines to obtain CETSA values in different cell lines, which is undoubtedly extremely expensive and time-consuming. With the help of CycleDNN, we only need to perform one CETSA biochemical experiment in one cell line (e.g.), HEK293T). The CETSA data for this protein in other cell lines will be predicted by CycleDNN instead of relying on experiments. CycleDNN has great advantages over traditional pair-wise DNN models (as shown in Fig. 4) for the prediction of CETSA data. First of all, due to the introduction of the common latent space *Z*, we significantly simplify the amount of parameters of the neural network. While maintaining the same size of the networks across *n* cell lines, our model reduces the number of encoders and decoders from $n(n-1)$ to *n*. In experiments of five cell lines, CycleDNN reduces the amount of model parameters by $75\%$. Moreover, our method also has a great advantage in prediction speed. In a typical task, we know the CETSA data for a new protein in one cell line and wish to predict the CETSA data in *n* other cell lines. Our method greatly reduces the number of encoders required in prediction, thereby increasing the prediction speed. In experiments of five cell lines, CycleDNN reduces the amount of encoder by $80\%$, which also reduces prediction time by $53.4\%$.

On the other hand, the proposed approach has two primary limitations. Firstly, the model necessitates initial training on the CETSA features of specific proteins found in both cell lines. It then predicts the CETSA features of the remaining proteins from one cell line to another. This capability is limited to handling CETSA feature translation only across cell lines used for training the model. However, the model cannot handle CETSA feature translation in cell lines that were not part of the training set. Secondly, while the proposed cycleDNN serves as an automated computational framework for predicting CETSA features across cell lines, and the predicted values closely align with the original experimental features as validated in this study, a thoughtfully designed biological evaluation is recommended to further confirm the biological significance of the predicted CETSA features.

## Conclusions

In this study, we focus on the transfer of CETSA data for the same protein across different cell lines, for which we propose a novel DNN model. The results of our proposed method, as applied to the protein melting data from intact cell MS-CETSA experiments, are presented in Tables 2,  3 and  4. The results demonstrate that it performs well in the prediction cross the cell lines A375, HCT116, HEK293T, HL60, and MCF7. The ablation study in Table 5 verifies the effectiveness of each of the three loss functions in our proposed model. At the same time, the neural architecture we design greatly reduces the complexity of the model from exponential to linear when converting CETSA features between different cell lines. Last but not least, we perform experiments using PPI predictions with predicted CETSA features from CycleDNN, which achieve similar performance compared to experimental CETSA features.

Our future research endeavors will focus on three key areas. Firstly, we aim to explore the potential utility of the encoded high-dimensional latent features in PPI prediction by comparing the performance of latent features extracted by CycleDNN and standard CETSA features. Secondly, we plan to extend the application of cycleDNN to different protein features. This will involve incorporating different types of features, such as protein amino acid sequences and structural attributes, into our model to enable the interconversion between different protein features. Lastly, a focal point will be the refinement of the network structure to improve the overall performance of our model and thereby expand its applicability in bioinformatics. Our goal is to develop a computational framework capable of seamlessly converting a broader range of protein data across different cell lines through a shared protein latent space.

### Supplementary Information

Supplementary Information.

## Data Availability

All the data generated or analyzed during this study are included in the supplementary information files.
